# Molecular Characterization and Phylogenetic Analysis of Feline Calicivirus Isolated in Guangdong Province, China from 2018 to 2022

**DOI:** 10.3390/v14112421

**Published:** 2022-10-31

**Authors:** Jianwei Mao, Shaotang Ye, Qi Li, Yumeizi Bai, Jieyan Wu, Liang Xu, Zhen Wang, Jingyu Wang, Pei Zhou, Shoujun Li

**Affiliations:** 1 College of Veterinary Medicine, South China Agricultural University, Guangzhou 510642, China; 2 Guangdong Provincial Key Laboratory of Prevention and Control for Severe Clinical Animal Diseases, Guangzhou 510642, China; 3 Guangdong Technological Engineering Research Center for Pet, Guangzhou 510642, China

**Keywords:** feline calicivirus, phylogenetic analysis, mutation, recombination, virulent systemic disease

## Abstract

Feline calicivirus (FCV) is a common feline infectious pathogen that mainly causes upper respiratory tract disease. To investigate the prevalence of FCV in Guangdong Province in China, a total of 152 nasal and throat swabs from cats suspected of FCV infection were collected in veterinary clinics or shelters from 2018 to 2022. The positive detection rate of FCV was 28.9% (44/152) by RT-PCR. In addition, twenty FCV isolates were successfully isolated and purified. Eleven out of twenty isolates were selected for further phylogenetic analyses based on the capsid protein VP1; our results revealed that seven isolates were in genogroup I, and four were in genogroup II. Notably, according to the whole genome phylogenetic tree, FCV-SCAU-11 was in the same branch as Korean isolates, and recombination analysis revealed that the FCV-SCAU-11 isolate showed potential recombinant events between the FCV-SH isolate and FCV-GXNN03-20 isolate. Furthermore, the virus replication kinetics indicated that FCV-SCAU-10, with clinically severe symptoms in patient cats, performed a more efficient replication in vitro. In conclusion, this study revealed the genetic diversity of FCVs in Guangdong Province, providing a reference for novel vaccine candidate strains and the development of effective strategies for preventing FCV infection in cats.

## 1. Introduction

Feline calicivirus (FCV) is now considered one of the major pathogens of cats worldwide. According to clinical symptoms, FCV can be divided into an upper respiratory tract disease (FCV-URTD) and virulent systemic disease (FCV-VSD). FCV-URTD has been associated with classical clinical signs and lesions, including respiratory disease, diarrhea, mucosal vesicular lesions, and ulcerations [1]. Besides the typical clinical symptoms of FCV-URTD, FCV-VSD include severe oral ulcers, limb edema, skin ulceration, bronchitis and pneumonia, and damage to the liver, pancreas, or spleen in some cases [2,3]. Reports of FCV-VSD have been gradually increasing worldwide [4,5,6]. Additionally, co-infection has been reported with feline herpesvirus 1 (FeHV-1), another major cause of upper respiratory tract disease in cats, and feline parvovirus (FPV), which induces panleucopenia and enteric symptoms [7]. Besides co-infection with other pathogens, many immunological failures and clinically recovered cats remain persistent infected carriers that contribute to the high prevalence of FCV in the cat population [8].

FCV is a non-enveloped single-stranded positive-sense RNA virus belonging to the *Vesivirus* in the family *Caliciviridae* [9]. The genome of FCV is about 7.7 kb and encodes three open reading frames (ORFs). ORF1 (about 20~5311 nucleotides) encodes polymeric non-structural proteins, which are hydrolyzed and cleaved into six non-structural proteins. In addition, Pro-Pol inhibits host gene expression and P39 can cleave host nucleotides [10]. ORF2 (about 5314~7320 nucleotides) encodes capsid protein VP1, which can be divided into six regions, A, B, C, D, E, and F, according to their amino acid sequence variability [11]. ORF3 (7217~7634 nucleotides) mainly encodes another capsid protein, VP2, which plays a vital role in FCV replication and viral particle assembly. It can also assist in the delivery of the FCV genome into its host cells [12].

The VP1 protein is the main structural protein of FCV and contains relatively conserved, as well as variable, regions. Therefore, phylogenetic analysis has traditionally been performed with gene VP1. According to the genetic diversity of the VP1 gene, FCV can be divided into two branches worldwide: genogroup I and genogroup II [13]. According to previous studies, most Chinese FCV isolates are clustered in genogroup II [14]. For decades, the principal preventive measure against FCV infection has been vaccination. However, the conventional vaccine fails to provide complete protection against the newer variant, FCV-URTD [15,16].

More FCV-VSD strains have been constantly emerging, and vaccination failure has frequently occurred in cases from recent years [17,18]. In order to provide a theoretical basis to grasp the epidemic situation and develop further measures for the prevention and control of FCV, this study investigates the epidemiological status and molecular characteristics of FCV in Guangdong Province, China.

## 2. Materials and Methods

### 2.1. Sample Information and Treatment

To monitor the FCV epidemic and its evolution, a total of 152 nasal and throat swabs were collected from cats suspected to be infected with FCV in veterinary clinics or shelters in Guangzhou, Yunfu, and Shenzhen in Guangdong Province from 2018 to 2022. Breed, age, sex, clinical symptoms, and vaccination status of the sampled cats were recorded in detail, as shown in Table 1. The samples were kept in sterile centrifuge tubes filled with Dulbecco’s modified Eagle medium (DMEM; Biological Industries, Kibbutz Beit-Haemek, Israel) and stored at −80 °C.

### 2.2. RT-PCR Screening for FCV

According to the manufacturer’s instructions, viral RNA was extracted from the collected samples by the OMEGA Mag-Bind Viral DNA/RNA Kit (Omega Bio-Tek, Norcross, GA, USA). Reverse transcription of cDNA was conducted using a HiScript III 1st Strand cDNA Synthesis Kit (Vazyme, Nanjing, China). A pair of specific primers were designed according to the conserved region of the FCV reference sequence obtained from the NCBI database. The forward primer was 5′-CTGCCTCCTACATGGGAAT-3′, and the reverse primer was 5′-GTGTATGAGTAAGGGTCRACCC-3′. The positive samples were then screened by a polymerase chain reaction using 2 × Rapid Taq Master Mix (Vazyme, Nanjing, China). The PCR reaction procedure was conducted as follows: pre-degeneration at 95 °C for 3 min; degeneration at 95 °C for 15 s; annealing at 55 °C for 15 s; extension at 72 °C for 30 s, with 35 cycles; and extension at 72 °C for 5 min. It was stored at 4 °C at the end of the reaction process.

### 2.3. Virus Isolation

The FCV-positive samples in PCR underwent filtration through a 0.45 μM filter membrane and were incubated with a Crandell Reese feline kidney (CRFK) cell monolayer and cultured in DMEM and 10% fetal bovine serum (Gibco, Grand Island, NY, USA) at 37 °C with 5% CO_2_ incubation. Inoculated cells were observed daily and harvested upon the appearance of cytopathic effect (CPE). All isolates were purified with three rounds of plaque purification. The plaque-purified virus was stored at −80 °C for further testing.

### 2.4. Whole Genome Sequencing

The whole genome of FCV-SCAU-1 to FCV-SCAU-11 were sequenced by next-generation sequencing (NGS). The NGS libraries of these ten isolates and assembling of whole genome were conducted as previously reported [6].

### 2.5. VP1 Gene Sequencing

A pair of primers was designed to amplify the full length of ORF2, according to the conserved region of a FCV reference sequence in the NCBI database. The forward primer was 5′-TTGAGCATGTGCTCAACCTG-3′ and the reverse primer was 5′-ATTTTGRTTTGTGTATGAGTAAGGG-3′. The VP1 gene of FCV-SVAU-12 to FCV-SVAU-21 was amplified with the following protocol: 95 °C for 3 min; 35 cycles of 95 °C for 30 s; 57 °C for 30 s; 72 °C for 2 min; and 72 °C for 10 min. PCR was performed with Phanta Super-Fidelity DNA Polymerase (Vazyme). Amplified DNA fragments were cut from the agarose gel and purified using a FastPure Gel DNA Extraction Mini Kit (Vazyme). The purified products were ligated into 5 min TA/Blunt-Zero Cloning vector (Vazyme). The positive recombinant plasmids were sequenced by Sangon (Sangon Biotech, Shanghai, China).

### 2.6. Sequence Analysis

A multiple-sequence alignment was constructed with the MAFFT software (version 7.313, Osaka, Japan), using the default settings [19]. Then, forty-two ORF2 and thirty-four full-length FCV genomes were downloaded from NCBI for phylogenetic analysis. Phylogenetic analyses were performed using MEGA software’s (version 11.0.11, Tempe, AZ, USA) maximum likelihood method [20]. To exclude the interference to phylogenetic clustering by close transmission events between individuals from the same premises, similar sequences of the capsid variable regions sharing greater than 76% sequence similarity were removed in our phylogenetic analysis [21]. Recombination is a common phenomenon in many RNA viruses [22]. In order to determine whether there were recombination events in the FCV isolates in Guangdong Province, all whole genomes of FCV sequences were downloaded from the NCBI database and aligned using MAFFT software. Then, they were initially screened for possible recombination events using RDP5 software (version 5.5, Martin DP, Cape Town 7549, South Africa) [23]. The recombination events were only considered when supported by at least three recombination detection algorithms, followed by an examination of the recombination signal, and estimating the location of the breakpoint using SimPlot software (version 3.5.1) (JHK University, Baltimore, MD, USA). The occurrence of recombination breakpoints was also confirmed by evolutionary trees drawn by the maximum likelihood method.

### 2.7. Indirect Fluorescence Assay

The monolayer of CRFK cells grown in a 6-well plate were inoculated with FCV. After 24 h, the cells were washed and then fixed in cold 4% paraformaldehyde at room temperature for 10 min. Then, cells were blocked for 10 min at room temperature (QuickBlock Blocking Buffer for Immunol Staining, Beyotime, Shanghai, China). After blocking, cells were incubated with the calicivirus monoclonal primary antibody (FCV1-43; Invitrogen, Waltham, MA, USA) overnight at 4 °C. After washing three times, cells were incubated with the Goat Anti-Mouse IgG H&L (Alexa Fluor^®^ 488; Abcam, Cambridge, UK) secondary antibody for 1 h at room temperature. After washing three times, nuclei were stained with 4′,6-diamidino-2-phenylindole (Beyotime, Shanghai, China) for 5 min, and then were washed three times. The fluorescence signal was captured by a fluorescence microscope (Leica, Wetzlar, Germany).

### 2.8. Transmission Electron Microscopy

CRFK cells were seeded into a 6-well cell plate. The monolayer cells were infected with FCV. At 24 h post-infection, cells were collected by centrifugation. The cell masses were fixed by a fixer for electron microscopy (Servicebio, Wuhan, China). After the fixation, the samples were processed according to the following procedure: dehydration, embedding, curing, block repair, sectioning, and staining. The dried sections were observed under the Talos L120C TEM (Thermo Fisher Scientific, Waltham, MA, USA).

### 2.9. Virus Replication Kinetics

Eleven isolates were selected for the evaluation of viral growth kinetics in CRFK cells. Analysis of the viral growth curve was performed according to the following procedure: the monolayer of CRFK cells grown in a 24-well plate were infected with FCV at a multiplicity of infection (MOI) of 0.01. The viruses were incubated for 1 h at 37 °C in 5% CO_2_. Next, the viruses were removed, and the cells were washed and replenished with fresh DMEM containing 2% FBS. At 4, 8, 12, 16, 20, and 24 h post-infection, the supernatant was harvested and stored at −80 °C until virus titer determination. Then, CRFK cells were grown to confluence in 96-well plates and inoculated with gradient dilutions of previous supernatant samples. Cells were incubated for 48 h at 37 °C. CPE was visually evaluated and compared with mock-inoculated cell monolayers under the bright field. Virus titer was calculated using the Reed and Muench method. All experiments were repeated three times.

### 2.10. Statistical Analysis

All data were analyzed with an unpaired Student’s t-test using Prism v9.3 (GraphPad Software, San Diego, CA, USA). (mean ± SD, * *p* < 0.05, ** *p* < 0.01, *** *p* < 0.001).

## 3. Results

### 3.1. Clinical Investigation and Virus Isolation

According to the RT-PCR results, there were forty-four FCV-positive samples in suspected cat cases. The overall FCV-positive rate was 28.9% (44/152). One of them potentially corresponded to a FCV-VSD strain (derived from a cat with severe clinical symptoms including high fever, limb edema, oral and skin ulceration, and pneumonia), whereas the others were isolated from cats with classical milder symptoms (FCV-URTD). Co-infection was found in our cases, with two positive tests for FPV and one positive test for FeHV-1. All the information on FCV isolates in this study is shown in Table 1. The subsequent virus isolation and identification were successful for 20 isolates; complete experiment details are described in the Supplemental Material.

### 3.2. Genetic and Phylogenetic Analysis of FCV Isolates

To compare the sequence identity of all the ten whole genome isolates (FCV-SCAU-1 to FCV-SCAU-11, except FCV-SCAU-6.), we blasted these sequences against each other and known sequences in the NCBI GenBank database. These sequences were aligned using MAFFT software, and a pairwise comparative analysis on nucleotides showed sequence identities ranging from 75.9 to 87.8%. We analyzed the nucleotide and amino acid sequence identities of FCV Guangdong isolates with FCV-255 (a widely used vaccine strain in China), based on three ORFs (Table 2). The ORF1 gene encodes six non-structural proteins with a length of 5292 nucleotides and a predicted protein precursor of 1763 amino acids. They showed a high identity match with strain FCV-225 in both nucleotides and amino acids. The length of VP1 genes in this study ranged from 2007 to 2010 nucleotides. They shared a low identity of 74.49–78.82% in nucleotides and 84.88–89.07% in amino acids with strain FCV-225. The ORF3 in this study shared the highest nucleotides and amino acids sequence identity with strain FCV-225 among the three ORFs. The twenty isolates differed in the length of the VP1 gene. The results showed that the VP1 capsid protein gene exhibited the highest variability within the FCV genome.

**Figure 1:**
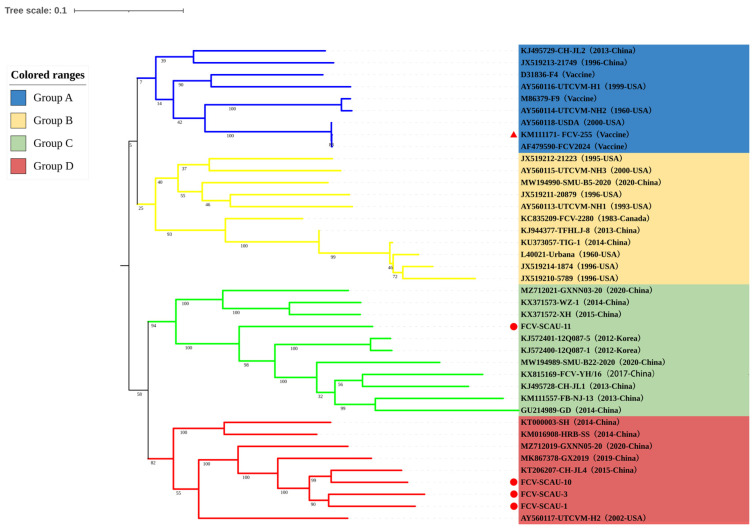
Phylogenetic trees were constructed based on the whole genome of FCV in the study. Evolutionary distances were computed using the Tamura-Nei model for 1000 bootstrap replicates. The sequences identified in the study were indicated with red circles and vaccine strains were marked with red triangles. The reference virus isolates are indicated by GenBank accession number, isolate name, and date and location of submission.

According to the whole genome phylogenetic tree, the FCV isolates could be separated into four groups (group-A, group-B, group-C, and group-D), and three FCV isolates were in the same group (group-D). The results showed that FCV-SCAU-1, FCV-SCAU-3, and FCV-SCAU-10 were in the same group with domestically prevalent isolates, CH-JL-4 (KT206207) from north China and GX2019 (MK867378) from south China. This suggested that FCV isolates in Guangdong Province have been circulating and evolving among feline populations between southern and northern China for a long time. Notably, FCV-SCAU-11 was in the same branch as Korean isolates, 12Q087-1 (2012-Korea) and 12Q087-5 (2012-Korea), and shared high homology. This suggested that FCV-SCAU-11 was widely spread in Korea and China (Figure 1).

**Figure 2:**
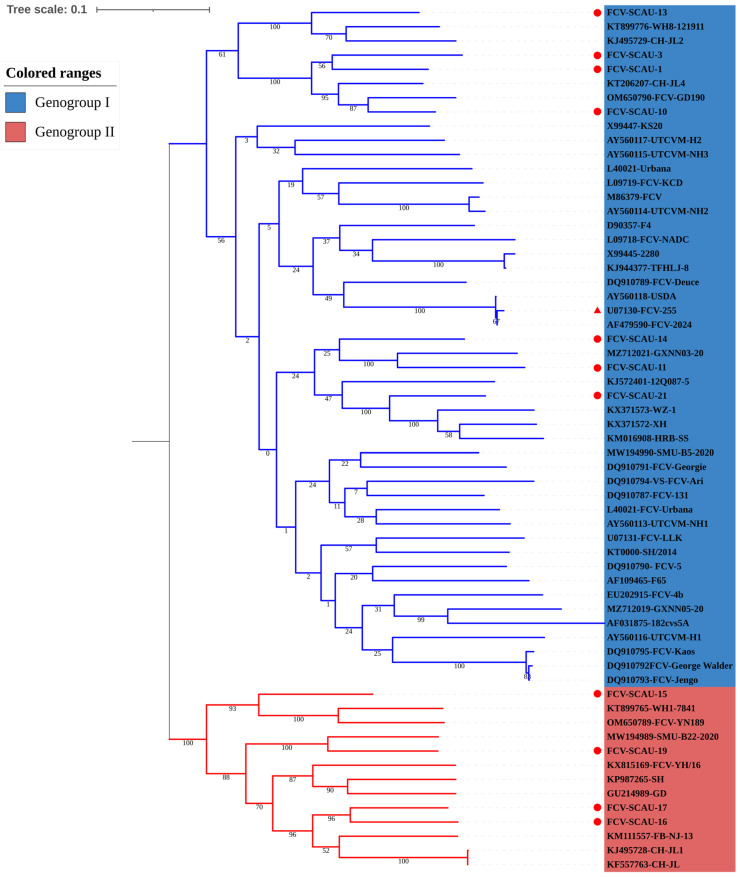
Phylogenetic trees were constructed based on the ORF2 in the study. The phylogenetic tree was constructed using the maximum-likelihood method for 1000 bootstrap values. The sequences identified in this study were labeled with red circles, and vaccine strains were labeled with red triangles.

The ML phylogenetic tree was constructed based on full-length ORF2 sequences of sixty-one FCV isolates, including eleven new isolates from Guangdong Province. FCV isolates from Guangdong Province were also clustered into two branches: genogroup I and genogroup II. Among the eleven FCV isolates in this study, seven were clustered in genogroup I, whereas the remaining four were clustered in genogroup II. The results suggested that the prevalent isolates in Guangdong Province were isolates of genogroup I. In addition, all isolates identified in the study were in a different cluster from the vaccine strain FCV-255 (Figure 2).

Taken together, the predominant isolates of FCV in Guangdong Province were mainly isolates of genogroup I. At the same time, the isolate (FCV-SCAU-11) shared high similarity to foreign isolates out of China.

### 3.3. Recombination Analysis of FCV Isolates

**Figure 3:**
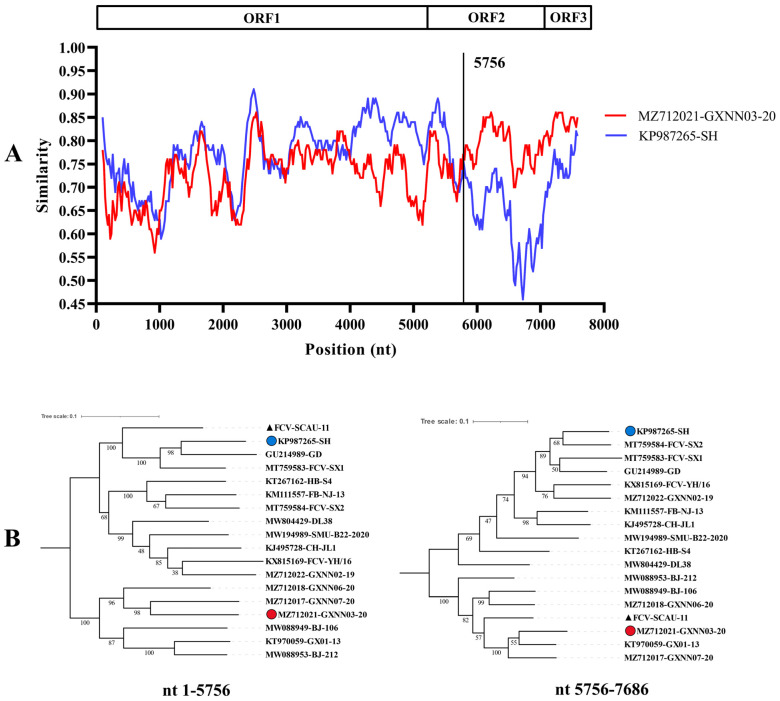
Recombination analysis of FCV-SCAU-11 identified in this study. (**A**) Genome scale similarity comparisons of FCV-SCAU-11 with FCV-SH isolate (blue) and FCV-GXNN03-20 isolate (red) using a sliding window (window size: 200 bp, step size: 20 bp). Potential recombination breakpoints were marked by a black vertical line with nucleotide sites at the bottom. (**B**) ML phylogenetic trees based on every recombinant fragment within FCV-SCAU-11and 15 reference FCV isolates are shown below the similarity plot. The isolate FCV-SCAU-11 is labeled with a black triangle, and the putative recombinant major parent isolate FCV-SH is marked with a blue circle. The putative recombinant minor parent isolate FCV-GXNN03-20 is marked with a red circle.

SimPlot and RDP5 software were used to identify possible recombinant events. The FCV-SCAU-11 isolate showed potential recombinant events between the FCV-SH and FCV-GXNN03-20 isolates with a *p*-value of <1.0 × 10^−6^ from the results of four detection methods (Table 3); more information about the viral strains used for comparison is described in Table S1. The positions of the recombination breakpoint in the FCV-SH and FCV-GXNN03-20 isolates were located in the VP1 gene at 5756 nucleotides in alignment. The breakpoints in FCV-SCAU-11 separated its genome into two regions, with regions A (nucleotides 1–5756) being closely related to the FCV-SH isolate, and region B (nucleotides 5756–7686) being closely related to the FCV-GXNN03-20 isolate. The recombination event was further confirmed by SimPlot and evolutionary trees were drawn using the maximum likelihood method (Figure 3).

### 3.4. Comparative Analysis of Amino Acids of the VP1 Proteins

The capsid proteins of FCV include VP1 encoded by ORF2 and VP2 encoded by ORF3. The capsid protein VP1 is the major capsid protein of FCV, encoded by 668 to 671 amino acid residues and can be divided into six regions, from A to F, according to their degree of amino acid variability. The region E (426–523 amino acid positions) in the capsid protein VP1 is hyper-variable, and can be separated by the conserved region (ConE) into two further regions (5′ HVR and 3′ HVR), which are responsible for antigenic variability, viral pathogenicity, and also involved with the cell receptor for initial attachment [24,25]. Most of the amino acid mutations in the capsid protein of FCV isolates from Guangdong Province occurred in the E region of 5′ HVR, which was consistent with previous studies. In this study, we found that FCV-SCAU-12, FCV-SCAU-16, and FCV-SCAU-19 each had three amino acids that were consistent with VSD strains (Table 4). However, these three FCV isolates showed no clinically severe symptoms in patient cats.

**Figure 4:**
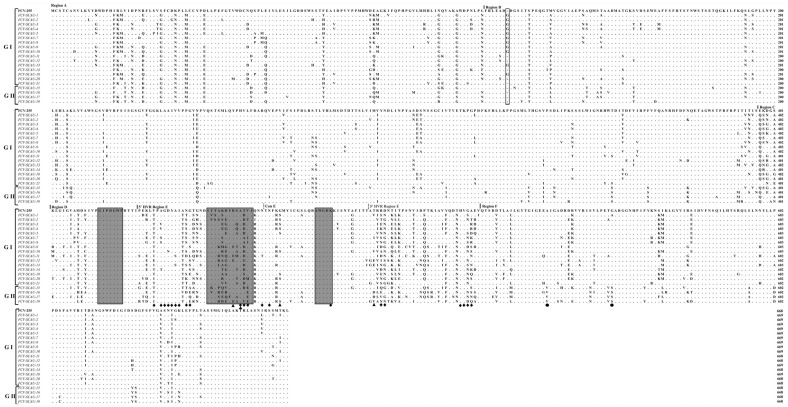
The alignment of the capsid sequences of ten FCV isolates and FCV-255 strain. The regions A, B, C, D, E, and F are indicated by vertical lines. A dashed line is used to divide the region E into 5′ HVR and 3′ HVR. The amino acid residues highly associated with VSD strains are marked with black triangles. The amino acid residues interacting between VP1 protein and fJAM-A receptor protein are marked with black diamonds. The deleted sites are indicated by hollow rectangle boxes.

Certain amino acids in VP1 can classify FCV isolates into genogroups I and II [26]. In this study, sixteen isolates of genogroup I possessed Asn/Lys/Asp, Ala, and Gly/Ala residues at positions 377, 539, and 557, respectively, whereas the remaining four isolates belonging to genogroup II had Lys, Val and Ser residues at positions 377, 539, and 557, respectively (Table S2). In addition, eleven isolates had an amino acid insertion at position 127 in the B region and the remaining nine isolates had a glycine at position 127 compared with the widely used vaccine strain FCV-255 in China (Figure 4). These mutations may also be responsible for changes in cell tropism of FCV [27].

The genetic markers distinguishing between VSD and URTD within the capsid protein remain inconclusive [28,29,30]. A recent study indicated that FCV-VSD and FCV-URTD strains differed in the physicochemical properties of the amino acids in region E (426–523), which contains the two hypervariable domains responsible for most of the antigenic variability and is involved in the binding to the FeJAM-1 receptor [31]. However, FCV-URTD strains in this study contained three consistent mutations associated with FCV-VSD, whereas the only FCV-VSD strain only shared two consistent mutations.

Overall, the potential relationship associated with the failure or deficiency of vaccination protection was unveiled. Moreover, there was no significantly recognizable evidence to differentiate between the FCV-URTD and FCV-VSD, at least in this study.

### 3.5. Virus Replication Kinetics of FCV Isolates

**Figure 5:**
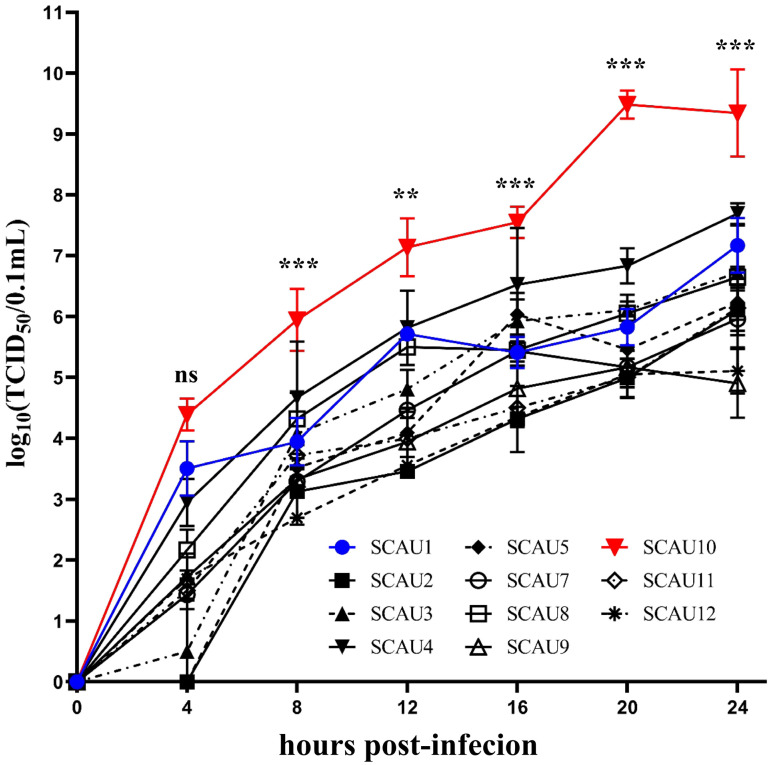
FCV growth kinetics monitored over 24 h in CRFK cells. Cells were inoculated with eleven isolates of FCV from Guangdong Province at 0.01 MOI. The FCV-SCAU-10 isolate is labeled with a red line and the FCV-SCAU-1 isolate is labeled with a blue line. Samples were analyzed in three independent experiments (ns indicated no significance, ** *p* < 0.01, *** *p* < 0.001).

The virus replication kinetics of eleven isolates at each time point was evaluated according to the Reed and Muench method. In the CRFK cells, the replication titer of the FCV-SCAU-10 isolate (on behalf of the VSD strain) was significantly higher than the FCV-SCAU-1 isolate (on behalf of the URTD strain) at all time points after infection. It reached a peak at 20 h post-infection. At this point in the virus titer, the average value of log10 (TCID50/0.1 mL) was 9.48 (Figure 5). The results suggested that FCV-SCAU-10 (a potential FCV-VSD strain) had more efficient replication in vitro.

## 4. Discussion

In this study, forty-two samples were positive for FCV, the overall FCV-positive rate was 28.9%, and twenty isolates were successfully isolated and purified from the positive samples. In addition, the results of whole genome analysis compared with other FCV isolates showed that FCV-SCAU-11 was in the same branch and shared high similarity with foreign strains out of China (strains in Korea). This suggested that a group of FCVs may be widely circulating in China and Korea due to geographic proximity. Further, FCV isolates from Guangdong Province were clustered into two branches, genogroup I and genogroup II, and most of the FCV Guangdong isolates were clustered in genogroup I (7/11), which is consistent with the latest studies (2021–2022) [14,27,32].

Among these FCV isolates, nineteen isolates were FCV-URTD, and one isolate was potentially FCV-VSD. A study reported that FCV-VSD strains had more efficient replication in cell culture and a broader spread in tissue culture than FCV-URTD strains [33]. In this study, the virus growth kinetics assay was carried out to compare the replication between FCV-URTD strains and FCV-SCAU-10 (potential VSD strain). The results showed that FCV-SCAU-10 had a significantly higher titer at all time points in CRFK cells than other FCV-URTD strains. This was consistent with a previous report that showed that the FCV-VSD strain efficiently replicated in vitro. However, an analysis of the genomic sequences of FCV-URTD and FCV-VSD is still not available to distinguish the different phenotypes. A recent hypothetical theory for differentiating between FCV-URTD and FCV-VSD indicated that seven amino acid mutations may lead to the different pathogenicity [31]. Unfortunately, subsequent studies have failed to fully support this theory, including the current study [6]. We found that a potential FCV-VSD strain in this study, FCV-SCAU-10, shared two consistent mutations in amino acid 465 and 492 of the VP1 capsid protein, whereas three FCV-URTD strains, FCV-SCAU-12, FCV-SCAU-16, and FCV-SCAU-19, each had three of the same amino acids mutations. This variance indicates an elusive mechanism differentiating between the FCV-UTRD and FCV-VSD. Two reports have successfully reproduced the experimental infection model with several independent FCV-VSD strains [30,34]. Due to the unclear and unique characterization of FCV-VSD strains, in vitro virus growth kinetic assays and in vivo experimental infection animal models may temporarily serve as the best method to distinguish between the FCV-URTD and FCV-VSD strains. However, the growth kinetics of FCV-SCAU-10 could not represent the remarkable characterization of all FCV-VSD strains. Further assays of FCV-SCAU-10 should be conducted, such as investigation of potential virus receptors, molecular pathogenicity, and animal infection experiments.

We noticed that some cats in this study with normal vaccination were still infected by FCV, which was consistent with previous studies [35,36]. This suggests that the vaccination failed to induce enough adaptive protection, which may be responsible for the limited effectiveness of vaccines. Therefore, we focused on analyzing variations in the major immunogenic antigen of FCV VP1 protein. The results of alignment of the VP1 sequences among the FCV isolates and FCV-255 vaccine strain showed the insertion of three consecutive G bases, encoding glycine at position 127 in region B. One study indicated that the spanning amino acids 445–451 (ITTANQY) of VP1 protein in the 3’ highly variable region was essential for neutralizing MAbs [37], and different amino acid residue mutations of the isolates were founded in this region. Linking to the vaccination status of patient cats, these changes may be responsible for differences in virulence and vaccination protection.

In conclusion, the overall prevalence of FCV infection was 28.9%, and we successfully isolated and purified twenty new FCV isolates from positive samples, including nineteen FCV-URTD strains and one potential FCV-VSD strain. In addition, the FCV isolates of Guangdong Province were from both genogroup I and genogroup II, mainly belonging to genogroup I. This study provided more valuable information on the genetic variation of the VP1 gene, which would be helpful in developing potential FCV vaccine candidate strains in the future.

## Figures and Tables

**Table 1:** Information of the FCV isolates in this study.

Location	Age (Months)	Sex	Clinical Symptoms	Vaccination Status	Isolation
Guangzhou	7	Female	Oral ulceration, salivation, conjunctivitis	No	FCV-SCAU-1
Guangzhou	8	Unknown	Unknown	No	FCV-SCAU-2
Guangzhou	4	Male	Eyes pus, salivation	No	FCV-SCAU-3
Guangzhou	5	Female	Oral ulceration, anorexia	No	FCV-SCAU-4
Guangzhou	Unknown	Female	Runny nose, conjunctivitis	No	FCV-SCAU-5
Shenzhen	8	Female	Coughing, conjunctivitis	No	FCV-SCAU-7
Shenzhen	24	Unknow	Coughing, conjunctivitis, salivation	Yes	FCV-SCAU-8
Guangzhou	Unknown	Female	Diarrhea, oral ulceration	No	FCV-SCAU-9
Guangzhou	12	Female	Oral and skin ulceration, limb edema, high fever, pneumonia	Yes	FCV-SCAU-10
Guangzhou	9	Female	Oral ulceration, salivation	Yes	FCV-SCAU-11
Yunfu	12	Female	Coughing, nasal eyelid edema	Yes	FCV-SCAU-12
Yunfu	13	Female	Coughing, conjunctivitis, salivation	Yes	FCV-SCAU-13
Yunfu	9	Female	Oral ulceration, conjunctivitis	Yes	FCV-SCAU-14
Yunfu	10	Female	Coughing, conjunctivitis, FPV positive	Yes	FCV-SCAU-15
Guangzhou	4	Male	Eyes pus, oral ulceration	No	FCV-SCAU-16
Guangzhou	5	Female	Coughing, oral ulceration, anorexia	No	FCV-SCAU-17
Guangzhou	4	Male	Oral ulceration, coughing, FPV positive	Yes	FCV-SCAU-18
Guangzhou	3	Male	Nasal eyelid edema, conjunctivitis	No	FCV-SCAU-19
Guangzhou	4	Male	Oral ulceration, FeHV-1 positive	No	FCV-SCAU-20
Guangzhou	5	Male	Runny nose, coughing, conjunctivitis	No	FCV-SCAU-21

**Table 2:** Homology analysis of three open reading frames (ORFs) of feline calicivirus (FCV) isolates compared with the 255 FCV strain.

				ORF1(%)			ORF2(%)			ORF3(%)	
Isolate	Pathotype	Genogroups	Length (nt/aa)	nt	aa	Length (nt/aa)	nt	aa	Length (nt/aa)	nt	aa
FCV-SCAU-1	URTD	I	5292/1763	79.77	92.23	2010/669	77.08	86.53	321/106	82.87	94.34
FCV-SCAU-2	URTD	I	5292/1763	80.00	92.57	2010/669	75.64	86.98	321/106	83.49	94.34
FCV-SCAU-3	URTD	I	5292/1763	78.80	91.77	2010/669	75.39	86.53	321/106	85.05	93.40
FCV-SCAU-4	URTD	I	5292/1763	78.78	91.77	2010/669	75.39	86.53	321/106	85.05	93.40
FCV-SCAU-5	URTD	I	5292/1763	80.40	91.71	2010/669	76.78	88.17	321/106	83.80	94.34
FCV-SCAU-7	URTD	I	5292/1763	79.47	92.51	2010/669	77.73	87.28	321/106	85.67	95.28
FCV-SCAU-8	URTD	I	5292/1763	79.41	92.29	2010/669	77.83	87.28	321/106	85.67	95.28
FCV-SCAU-9	URTD	I	5292/1763	79.24	92.06	2010/669	77.73	87.43	321/106	86.29	95.28
FCV-SCAU-10	Potential VSD	I	5292/1763	80.00	92.17	2010/669	77.08	87.72	321/106	84.42	93.40
FCV-SCAU-11	URTD	I	5292/1763	77.60	89.60	2007/668	76.73	85.93	321/106	84.11	95.28
FCV-SCAU-12	URTD	I	-	-	-	2007/668	75.64	87.28	-	-	-
FCV-SCAU-13	URTD	I	-	-	-	2010/669	76.23	86.83	-	-	-
FCV-SCAU-14	URTD	I	-	-	-	2007/668	78.82	88.77	-	-	-
FCV-SCAU-15	URTD	II	-	-	-	2007/668	76.68	86.08	-	-	-
FCV-SCAU-16	URTD	II	-	-	-	2007/668	74.49	85.03	-	-	-
FCV-SCAU-17	URTD	II	-	-	-	2007/668	75.34	85.48	-	-	-
FCV-SCAU-18	URTD	I	-	-	-	2010/669	76.38	87.57	-	-	-
FCV-SCAU-19	URTD	II	-	-	-	2007/668	75.09	84.88	-	-	-
FCV-SCAU-20	URTD	I	-	-	-	2007/668	77.43	86.08	-	-	-
FCV-SCAU-21	URTD	I	-	-	-	2007/668	78.23	89.07	-	-	-

**Table 3:** Information on recombination events of FCV-SCAU-11 detected by RPD5 software.

Recombinant	Major (Similarity)	Minor (Similarity)	*p*-Value of the Detection Methods						
			RDP	GENECONV	BootScan	MaxChi	Chimaera	SiScan	3Seq
FCV-SCAU-11	FCV-SH(94.3%)	FCV-GXNN03-20(98.4%)	9.829 × 10^−11^	NS	NS	2.405 × 10^−8^	5.319 × 10^−11^	3.247 × 10^−14^	NS

Note. NS indicates no significance.

**Table 4:** Comparative amino acids of region E of FCV capsid proteins with VSD strains.

Genogroup	Isolate	Amino-Acid Residues and Physico-Chemical Properties Associated with VSD Pathotype						
		438	440	448	452	455	465	492
	VSD-FCV	V	Q	K/R	E	T	S	V
	ORO-FCV	T	G	A	D	D	G	V
GI	FCV-SCAU-1	T	G	A	D	D	**S**	I
GI	FCV-SCAU-2	T	G	S	D	D	**S**	**V**
GI	FCV-SCAU-3	T	G	A	D	D	**S**	I
GI	FCV-SCAU-4	T	G	A	D	D	**S**	I
GI	FCV-SCAU-5	T	G	A	D	D	**S**	**V**
GI	FCV-SCAU-7	T	G	A	D	A	G	**V**
GI	FCV-SCAU-8	T	G	A	D	A	G	**V**
GI	FCV-SCAU-9	T	S	**K**	**E**	D	G	I
GI	FCV-SCAU-10	T	G	A	D	D	**S**	**V**
GI	FCV-SCAU-11	T	L	**R**	**E**	D	G	I
GI	FCV-SCAU-12	T	S	**R**	**E**	G	G	**V**
GI	FCV-SCAU-13	T	S	L	D	D	G	I
GI	FCV-SCAU-14	T	S	A	D	D	G	**V**
GI	FCV-SCAU-18	T	E	A	D	S	**S**	**V**
GI	FCV-SCAU-20	T	G	A	D	D	**S**	**V**
GI	FCV-SCAU-21	T	G	A	D	D	G	**V**
GII	FCV-SCAU-15	T	A	P	D	D	G	I
GII	FCV-SCAU-16	**V**	E	**R**	D	**T**	G	L
GII	FCV-SCAU-17	T	**Q**	S	D	**T**	G	L
GII	FCV-SCAU-19	T	E	**R**	**E**	V	**S**	A

Note. Grey-shaded bold font indicates amino acid residues highly associated with VSD strains, referred to in previous reports.

## Data Availability

The data that support the findings of this study are available from the corresponding author upon reasonable request.

## Supplementary Materials

The following supporting information can be downloaded at: https://www.mdpi.com/article/10.3390/v14112421/s1, Supplementary File S1: The sequences of FCV isolated in this study; Figure S1: Isolation and identification of FCV in this study; Table S1: Information of two isolates used for recombination analysis; Table S2: Differences in certain amino acids of Genogroups I and II.
