# Supplementary material for: Molecular Characterization and Phylogenetic Analysis of Feline Calicivirus Isolated in Guangdong Province, China from 2018 to 2022
Source: Viruses. 2022 Oct 31;14(11):2421. doi: 10.3390/v14112421 (PMC9696216; doi:10.3390/v14112421)
Supplement: Supplementary file 1 [file viruses-14-02421-s001.zip › Figure S1. Isolation and identification of FCV in this study.pdf]

Article

Molecular Characterization and Phylogenetic Analysis of Feline Calicivirus Isolated in Guangdong Province, China from 2018 to 2022

Jianwei Mao [1,2,3,†], Shaotang Ye [1,2,3,†], Qi Li [1,2,3,†], Yumeizi Bai [1,2,3], Jieyan Wu [1,2,3], Liang Xu [1,2,3], Zhen Wang [1,2,3], Jingyu Wang [1,2,3], Pei Zhou [1,2,3,*] and Shoujun Li [1,2,3,*]

1 College of Veterinary Medicine, South China Agricultural University, Guangzhou 510642, China
2 Guangdong Provincial Key Laboratory of Prevention and Control for Severe Clinical Animal Diseases, Guangzhou 510642, China
3 Guangdong Technological Engineering Research Center for Pet, Guangzhou 510642, China
* Correspondence: zhoupei@scau.edu.cn (P.Z.); shoujunli@scau.edu.cn (S.L.); Tel.: +86-13826481597 (P.Z.); +86-13503030878 (S.L.)
† These authors contributed equally to this work.

1. Isolation and Identification of FCV from Clinical Samples

The clinical samples that tested positive by PCR were incubated with CRFK cells. The cytopathic effect appeared in cells within five days. All isolates underwent three rounds of plaque purification, and twenty FCV isolates were successfully isolated and named as FCV-SCAU-1 to FCV-SCAU-21 (except FCV-SCAU-6). It was worth noticing that FCV-SCAU-6 was abandoned here because of the unexpected bacteria contamination. These twenty isolates were incubated with CRFK cells, then indirect fluorescence analysis specific for FCV were carried out. The IFA results show that specific green fluorescence could be observed under the fluorescence microscope, while there was no green fluorescence in the negative control group. In addition, the typical virions of FCV with a diameter of approximately 30 nm were observed in infected CRFK cells with transmission electron microscopy.

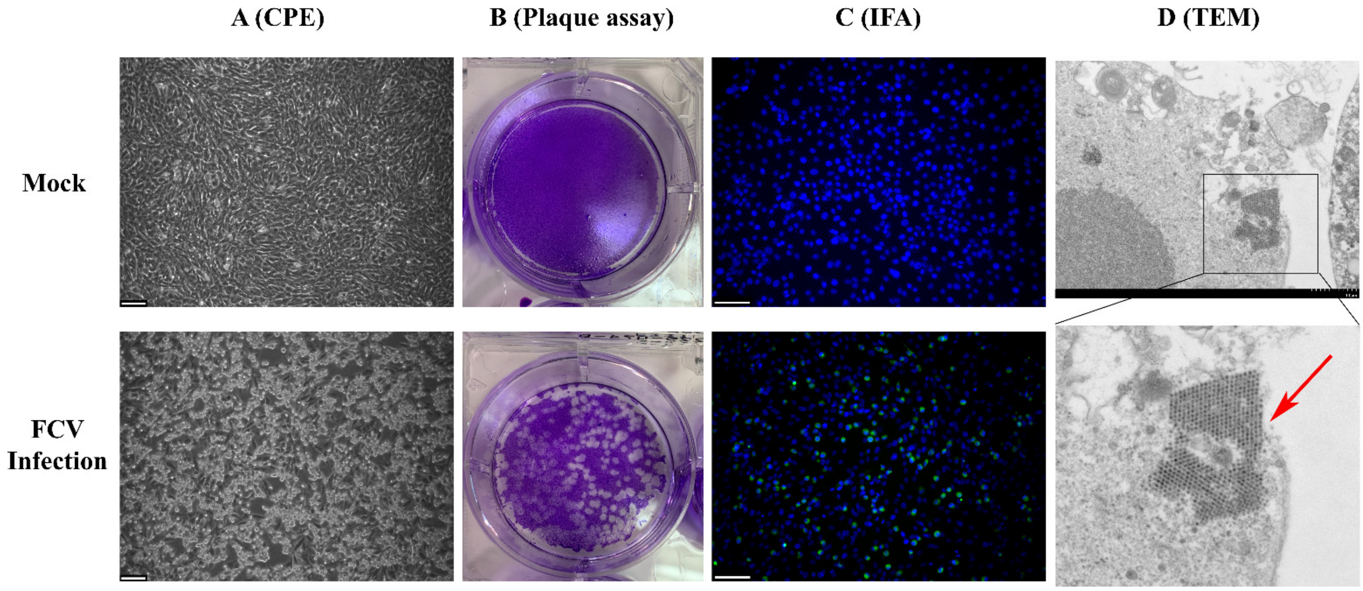

Figure S1. Isolation and identification of FCV in this study. (A) CPE was shown in CRFK cells inoculated with FCV-SCAU-1 or the uninfected control at 12 h post-infection. Scale bar, 50 μm; (B) Plaque assay for FCV-SCAU-1 isolate in CRFK cells; (C) Indirect fluorescence assay (IFA) of CRFK cells infected with FCV-SCAU-1 isolate and uninfected medium at 12 h post-infection. Scale bar, 75

μm; (D) Feline calicivirus (FCV-SCAU-3) particles are observed in cell by transmission electron microscopy (TEM). Red arrows indicated the FCV particles. Scale bar, 1 μm.
