# Supplementary material for: Molecular Characterization and Phylogenetic Analysis of Feline Calicivirus Isolated in Guangdong Province, China from 2018 to 2022
Source: Viruses. 2022 Oct 31;14(11):2421. doi: 10.3390/v14112421 (PMC9696216; doi:10.3390/v14112421)
Supplement: Supplementary file 1 [file viruses-14-02421-s001.zip › Table S1. Information of two isolates used for recombination analysis .pdf]

Table S1. Information of two isolates used for recombination analysis

Strains	Accession number	Whole genome(nt) PolyA tails removed	Isolation year	Isolation site
FCV-SH	KP987265	7684	2014	Jilin, China
FCV-GXNN03-20	MZ712021	7688	2020	Guangxi, China
