# Supplementary material for: Molecular Characterization and Phylogenetic Analysis of Feline Calicivirus Isolated in Guangdong Province, China from 2018 to 2022
Source: Viruses. 2022 Oct 31;14(11):2421. doi: 10.3390/v14112421 (PMC9696216; doi:10.3390/v14112421)
Supplement: Supplementary file 1 [file viruses-14-02421-s001.zip › Table S2. Differences in certain amino acids of Genogroups I and II.pdf]

Table S2. Differences in certain amino acids of Genogroups I and II

Genogroup	Isolates	377	539	557
I	FCV-SCAU-1	N	A	A
I	FCV-SCAU-2	K	A	G
I	FCV-SCAU-3	N	A	G
I	FCV-SCAU-4	N	A	G
I	FCV-SCAU-5	N	A	G
I	FCV-SCAU-7	N	A	G
I	FCV-SCAU-8	N	A	G
I	FCV-SCAU-9	N	A	G
I	FCV-SCAU-10	N	A	G
I	FCV-SCAU-11	N	A	G
I	FCV-SCAU-12	D	A	G
I	FCV-SCAU-13	N	A	G
I	FCV-SCAU-14	N	A	G
I	FCV-SCAU-18	N	A	G
I	FCV-SCAU-20	N	A	G
I	FCV-SCAU-21	N	A	G
I I	FCV-SCAU-15	K	V	S
I I	FCV-SCAU-16	K	V	S
I I	FCV-SCAU-17	K	V	S
I I	FCV-SCAU-19	K	V	S
